# 

**Published:** 1908-07-18

**Authors:** 


					rtT* *r
' TTtt^ MODERN
T*logr?phlo Address I , . . . m 2734 Gerrard.
Oapednle, London. o L jJOXJR-NAL*
HEW  -Sattodax
JxiLY 18th, 1908.
PRICE THREEPENCE.

				

